# Association between Estrogen Receptor-*α* Gene XbaI and PvuII Polymorphisms and Periodontitis Susceptibility: A Meta-Analysis

**DOI:** 10.1155/2015/741972

**Published:** 2015-11-24

**Authors:** Hong Weng, Chao Zhang, Yuan-Yuan Hu, Rui-Xia Yuan, Hong-Xia Zuo, Jin-Zhu Yan, Yu-Ming Niu

**Affiliations:** ^1^Center for Evidence-Based Medicine and Clinical Research, Taihe Hospital, Hubei University of Medicine, Shiyan 442000, China; ^2^Institute of Medicine and Nursing, Hubei University of Medicine, Shiyan 442000, China; ^3^Department of Stomatology, Taihe Hospital, Hubei University of Medicine, Shiyan 442000, China

## Abstract

*Background*. Certain studies have previously explored the association between the estrogen receptor-*α* (ER-*α*) gene polymorphisms and periodontitis susceptibility, although the current results are controversial. The present study, using meta-analysis, aimed to investigate the nature of the genetic susceptibility of the ER-*α* for developing periodontitis.* Methods*. A comprehensive literature search of PubMed, Embase, CNKI, and Wanfang databases was conducted up to January 8, 2015. Statistical manipulation was performed using Stata version 13.0 software. Odds ratios (ORs) and corresponding 95% confident intervals (CIs) were calculated to estimate the association in five genetic models.* Results*. A total of 17 eligible case-control studies from seven identified publications consisting of nine studies for the XbaI polymorphism and eight studies for the PvuII polymorphism were included in the meta-analysis. We found elevated risk of periodontitis in XbaI XX genotype carriers. Moreover, subgroup analyses demonstrated increased risk for chronic periodontitis of XbaI XX genotype carriers, specifically in the Chinese Han female population. No significant association was observed between PvuII polymorphism and periodontitis.* Conclusion*. Current evidence indicated that the homozygote (XX) genotype of ER-*α* gene XbaI polymorphism, but not PvuII mutation, may increase the risk of chronic periodontitis, specifically in the Chinese Han female population.

## 1. Introduction

Periodontitis, one of the most common chronic inflammatory diseases, is a chronic infectious disease of the supporting tissues of the teeth [1, 2]. Periodontitis is considered a complex and multifactorial disease that has a relatively mild phenotype and is chronic and tardily progressive in nature [3]. It is a major cause of tooth loss in adults: bacterial infection that the teeth lose their ligamentous support [2, 4, 5]. The amount of bacterial plaque and species do not indispensably relate with disease severity, while oral bacteria are essential for the initiation of periodontitis [6, 7]. Each person presents an individual dose-response curve to bacterial infection, which defines the susceptibility of the host to periodontitis [8]. Certain individuals are disease-resistant and will not develop periodontitis. Today, the etiology of periodontitis, which consists of both genetic and environment factors, such as smoking and stress, remains unclear [9]. A considerable number of scientific papers have explored the vital role of genes and their corresponding mutations in host responses and in disease progression [10–16].

Osteoporosis is a disease of the skeletal system characterized by reductions in bone mass and leading to increased bone fragility and facture. Both osteoporosis and periodontitis are bone destructive diseases; thus osteoporosis has been assumed to be a risk factor for developing periodontitis [17]. Several studies have reported a positive association between osteoporosis and periodontitis [18]. Certain individual factors related to bone metabolism may contribute to the progression and susceptibility of periodontitis [19]. A previous meta-analysis has suggested that vitamin D receptor (VDR) gene Taq-I and Fok-I polymorphisms may increase chronic periodontitis, specifically in Asians [20]. To the best of our knowledge, estrogen is one of the hormones regulating bone metabolism and its receptor is estrogen receptor (ER), which presents as two isoforms, namely, ER-*α* and ER-*β* [19]. Some evidence shows that ER-*α* gene XbaI and PvuII polymorphisms are associated with bone mineral density and osteoporosis [21, 22]. Therefore, a relationship between ER polymorphisms and periodontitis susceptibility may exist. Several studies have also investigated the association between ER-*α* and periodontitis susceptibility; however, the results obtained are fairly controversial [19, 23–28]. Discrepancies observed between individual studies may be caused by low statistical power, small sample sizes, and clinical heterogeneity. Therefore, to resolve the limitations of individual research, meta-analysis was performed in the present work. The objective of this meta-analysis is to clarify whether or not ER-*α* gene XbaI and PvuII polymorphisms are associated with periodontitis susceptibility.

## 2. Materials and Methods

This meta-analysis follows the Meta-analysis of Observational Studies in Epidemiology (MOOSE) statement [29].

### 2.1. Search Strategy

We searched the PubMed, Embase, CNKI, and Wanfang databases up to January 8, 2015, to collect studies that examined the association between the ER-*α* polymorphisms and periodontitis with the following search items: [(periodontitis OR periodontal disease) AND estrogen receptor AND polymorphism]. Reference lists of the identified studies and recent reviews were retrieved for additional studies.

### 2.2. Eligible Criteria

Studies were identified in the meta-analysis if they met the following criteria: (1) the study design was case-control or cohort; (2) the study examined the association between the ER-*α* polymorphisms and periodontitis susceptibility; (3) the study included patients who were diagnosed with periodontitis and controls who were periodontally healthy; (4) the study included sufficient data for genotype distribution to calculate odds ratios (ORs) and corresponding 95% confidence intervals (CIs); (5) the study was published in Chinese or English.

We excluded the following: (1) studies with overlapping data or duplicate publications; (2) the genotype distributions that could not be ascertained or calculated; (3) the study subject who was not human.

### 2.3. Data Extraction

Two researchers (Hong Weng and Chao Zhang) extracted the following trial information from qualified publications: author, publication year, country, ethnicity of the study population, type of disease, source of DNA sample and control, genotyping method, age of study subjects, demographics, number of cases and controls for each ER-*α* gene XbaI and PvuII genotype, genotype distribution, Hardy-Weinberg equilibrium (HWE) for controls, and related information for study quality assessment [30]. Additionally, any disagreements were resolved by discussion between the two authors. The *κ* test was used to assess interexaminer consistency (*P* = 0.95).

### 2.4. Quality Assessment

Two authors (Yuan-Yuan Hu and Rui-Xia Yuan, *P* = 0.93 for *κ* test) independently assessed the quality of the included studies using quality scoring criteria modified from previous meta-analyses (see Supplementary Table 1 in Supplementary Material available online at http://dx.doi.org/10.1155/2015/741972) [31–33]. This modified criterion was based on traditional quality scoring for observational studies and took both traditional epidemiological matters and genetic issues into consideration. Quality scores ranged from 0 to 11 points. Studies scoring seven points or higher were considered of high quality, while those scoring less than seven points were classified as low quality [32].

### 2.5. Statistical Analysis

The chi-squared test was used to assess whether the genotype frequencies in controls were consistent with HWE. The ORs and relevant 95% CIs were calculated to quantify the strength of association between the ER-*α* gene XbaI and PvuII polymorphisms and periodontitis susceptibility using five genetic models: allelic contrast, homozygote contrast, heterozygote contrast, dominant model, and recessive model. The effect of heterogeneity was estimated using the *I* ^2^ statistic [34]. The random-effect model was employed to pool the results of the included studies in the presence of significant between-study heterogeneity (value of *I* ^2^ ≥ 50% and *P* ≤ 0.1); otherwise, the fixed-effect model was utilized. Stratification analyses based on the type of disease, HWE status for controls, ethnicity, and gender were also performed. Publication bias was assessed by funnel plot and Egger's linear regression test to quantitatively measure the asymmetry of funnel plots [35]. We employed the step-down Bonferroni and Benjamini-Hochberg methods to control the false discovery rate (FDR) and adjust for multiple statistical comparisons [36, 37]. The power of individual studies was further calculated at a significance level of 0.05, and an OR equaling 1.5 was considered a small effect size [38]. Meta-analysis and the HWE test were conducted using Stata 13.0 software, and power analyses were conducted using G^*∗*^Power statistical software.

## 3. Results

### 3.1. Study Selection and Characteristics

**Figure 1:**
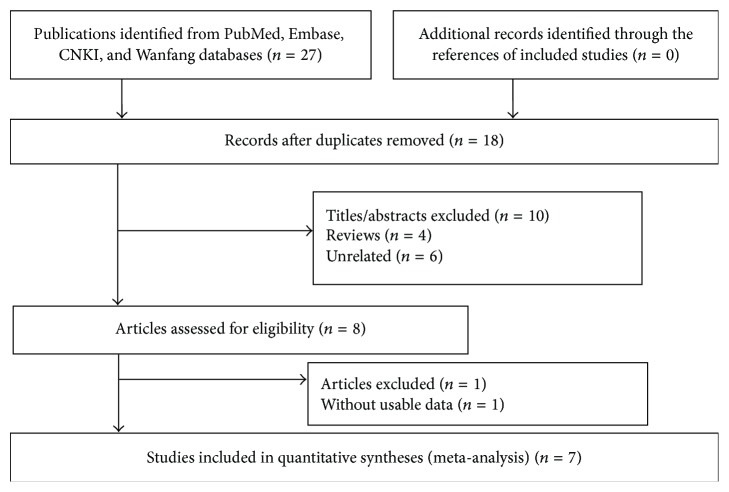
Flowchart for study section of this meta-analysis.

Twenty-seven studies were retrieved by a systematic literature search, and eight were selected for a full-text review after screening of their title and abstract details [19, 23–28, 39]. One study was excluded because it was without usable data [39]. Ultimately, seven studies satisfied our inclusion criteria [19, 23–28], and a total of 17 eligible case-control studies consisting of nine studies [19, 23–28] for the XbaI polymorphism containing 757 cases and 827 controls and eight studies [19, 23, 25–28] for the PvuII polymorphism containing 651 cases and 747 controls were obtained from these publications. All of the studies focused on the Chinese population: one publication [28] studied both the Chinese Han and Hui populations, while the rest of the studies included only the Chinese Han population. Genotyping of polymerase chain reaction fragment length polymorphisms was performed in all of the included studies (data not shown). The statistical powers of these 17 studies ranged from 17.89% to 38.14%, and quality scores ranged from seven to nine points, with a mean score of 7.8 points.  Figure 1 shows the study selection process; Table 1 summarizes the baseline characteristics and methodological quality of the included studies.

### 3.2. Overall Analyses

**Figure 2:**
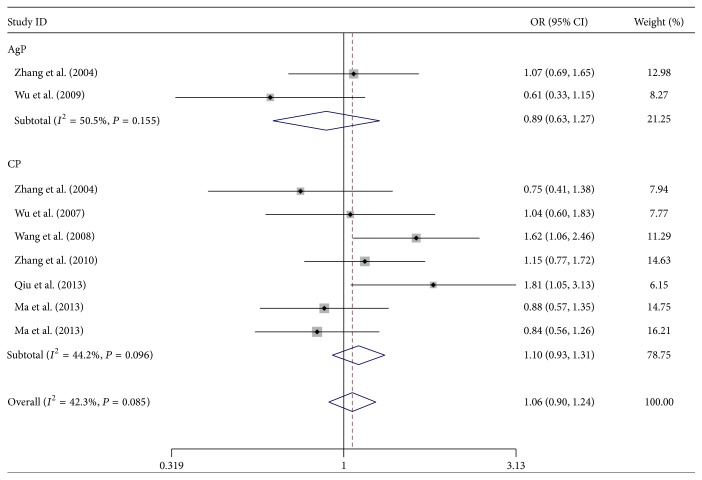
Forest plot for estrogen receptor-*α* gene XbaI polymorphism associated with risk of chronic and aggressive periodontitis in X versus x allele comparison in all study subjects.

Tables 2 and 3 illustrate the results of overall and subgroup analyses for ER-*α* XbaI and PvuII polymorphisms, respectively. In general, elevated risk of periodontitis was found in XbaI XX genotype carriers (XX versus xx: OR = 1.67, 95% CI = 1.13 to 2.47, *P* = 0.010, and FDR = 0.025 with *P* = 0.040 for step-down Bonferroni testing, Figure 2; XX versus Xx+xx: OR = 2.07, 95% CI = 1.43 to 3.00, *P* = 0.001, and FDR = 0.005 with *P* = 0.005 for step-down Bonferroni testing); such risk, however, was not significant in three other genetic models (X versus x: OR = 1.06, 95% CI = 0.90 to 1.24, and *P* = 0.480; Xx versus xx: OR = 0.72, 95% CI = 0.51 to 1.03, and *P* = 0.074; XX+Xx versus xx: OR = 0.88, 95% CI = 0.72 to 1.07, and *P* = 0.202) (Table 2). No significant association was observed between the ER-*α* PvuII polymorphism and periodontitis susceptibility (Table 3).

### 3.3. Subgroup Analyses

When results of the subgroup analyses for ER-*α* XbaI polymorphism were stratified by HWE status of controls, type of disease, ethnicity, and gender, significant associations were observed among the subgroup in which controls are consistent with HWE, chronic periodontitis, female population, and the Chinese Han population; these findings are similar to the overall analysis results of homozygote contrast (XX versus xx) and the recessive genetic model (XX versus Xx+xx) (Table 2). Decreased risk of periodontitis in Xx genotype carriers was observed when data were extracted according to gender (Table 2). The results of subgroup analyses for ER-*α* PvuII polymorphism are similar to those of the overall analyses (Table 3).

### 3.4. Publication Bias

**Figure 3:**
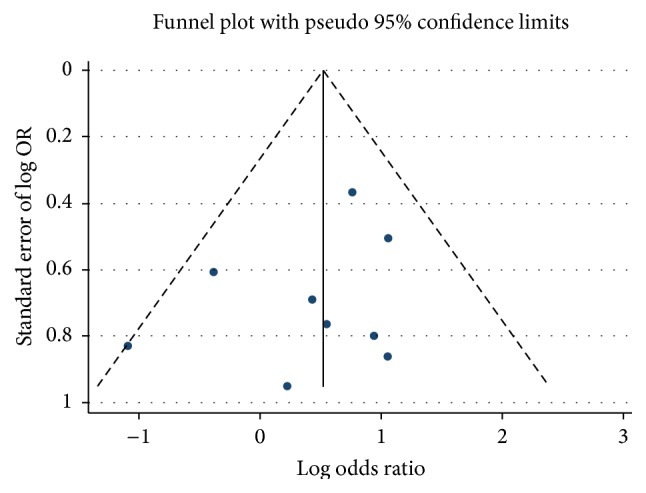
Funnel plot for publication bias for estrogen receptor-*α* gene XbaI polymorphism based on X versus x allele model.

A funnel plot based on the X versus x genetic model showed a relatively symmetrical distribution, which indicates that no publication bias exists in the present meta-analysis (*P* = 0.492 for Egger's linear regression test) (Figure 3).

## 4. Discussion

Periodontal disease, which is related to many other diseases [40–42], presents escalating burdens to the healthcare economy [43, 44]; therefore, determining the etiology and/or risk factors of periodontitis is necessary to prevent and cure the disease. The complex and multifactorial nature of periodontitis is well-acknowledged, and genetic factors are considered to be strongly associated with the disease. A number of researchers have identified and evaluated the role of genes and relevant mutations in the initiation and progression of periodontitis.

In the present meta-analyses, we pooled results from published studies to examine associations between ER-*α* XbaI and PvuII polymorphisms and risk of periodontitis. A total of 757 patients and 827 controls for the XbaI polymorphism and 651 patients and 747 controls for the PvuII polymorphism were included in our meta-analysis. All of the studies were carried out in China. Results of the overall population revealed that XbaI XX homozygotes present a roughly 1.67-fold higher risk of developing periodontitis than subjects with the xx genotype and 2.07-fold higher risk than carriers with Xx and xx genotypes. These results are supported by the fact that only low heterogeneity was observed between studies. Xx heterozygotes showed decreased risk of periodontitis compared with the xx genotype, but differences between these groups did not show statistical significance. Subgroup analyses based on the type of periodontitis showed that XX-genotype carriers present increased risk of chronic periodontitis instead of aggressive periodontitis. A significant association was observed between the Chinese Han population and periodontitis but not in the Hui population stratified by ethnicity. However, since the results of the Hui population were based on only one study, findings must be interpreted with caution. When the data were analysed based on gender, female XX genotype carriers appeared to show increased risk of periodontitis compared to male XX genotype carriers. Xx genotype carriers appeared to show decreased risk of periodontitis (Table 2). We thus hypothesized that the nonsignificant association of the overall analysis in the dominant genetic model (XX+Xx versus xx) may be attributed to the protective effect of the heterozygote genotype, which may decrease susceptibility to periodontitis. Determination of the final association of Xx heterozygotes with the XbaI polymorphism warrants further study.

Periodontal disease is a bone destructive disease; thus, certain individual factors related to bone metabolism may contribute to the progression and susceptibility of periodontitis [17, 19]. As such, several studies have been conducted to examine the association between ER-*α* gene polymorphisms and periodontitis susceptibility. The disease is complex and multifactorial; hence, the results of epidemiological studies are usually inconsistent [10, 16]. The present meta-analysis is a useful summary of current evidence highlighting the association between ER-*α* gene polymorphisms and risk of periodontitis and provides clinical evidence to help clinicians and researchers prevent, diagnose, and cure periodontitis. We note, however, that only the relations of ER-*α* XbaI and PvuII polymorphisms to periodontitis risk are discussed in this meta-analysis; other polymorphism variants may present relations with periodontitis risk and should also be examined in further research.

The retrospective meta-analysis presents several limitations that must be taken into consideration. First, the case-control studies included in this meta-analysis were of small or medium sample size; thus, they may have low statistical power for investigating final associations. Second, six of the identified case-control studies for ER-*α* XbaI polymorphism included controls that were not consistent with the HWE. Deviation from the HWE in controls suggests certain biases during control selection and/or genotyping errors [45]. Third, the subjects in this meta-analysis were mostly of Chinese Han descent and only one study focused on the Chinese Hui population; hence, our results are only applicable to these ethnic populations because the number and type of genes influencing certain disease may not be identical in diverse populations. Fourth, confounding factors, such as smoking and other types of genetic interplay, may distort the result of this meta-analysis because of insufficient data. Publication bias cannot be completely eliminated regardless of the funnel plot and Egger's linear regression test results, which may also influence the results of the meta-analysis. Finally, while the ER-*α* gene XbaI polymorphism may be associated with the severity of periodontitis, insufficient information does not allow us to determine the associations between ER-*α* gene polymorphisms and severity of periodontitis.

## 5. Conclusion

In summary, our meta-analysis indicated that the homozygote (XX) genotype of ER-*α* gene XbaI polymorphism may increase the risk of chronic periodontitis, specifically in the Chinese Han female population. But the PvuII polymorphism may not be associated with periodontitis. Large scale and high quality studies are necessary to validate the risk recognized in this meta-analysis.

## Supplementary Material

The scale for quality assessment is in supplementary Table 1.

## Figures and Tables

**Table 1:** Characteristics of the studies included in the present meta-analysis.

Authors and year	Race	Disease type	DNA extraction method	Control source	Age (years)^*∗*^	Sample size^*∗*^	Females (%)^*∗*^	*P* for HWE	Power (%)^†^	Quality score
XbaI (rs9340799)										
Zhang et al., 2004 [19]	Han	AgP	Peripheral blood	PB	29.0 ± 6.3/29.1 ± 6.2	90/91	58.9/52.8	<0.01	26.97	7
Zhang et al., 2004 [19]	Han	CP	Peripheral blood	PB	38.0 ± 10.4/29.1 ± 6.2	34/91	44.1/52.8	<0.01	20.00	7
Wu et al., 2007 [23]	Han	CP	Buccal mucosa	HB	49/35	55/60	56.4/50.0	0.04	18.76	8
Wang et al., 2008 [24]	Han	CP	Peripheral blood	HB	41.7 ± 7.4/40.8 ± 6.0	106/80	59.4/60.0	0.01	27.59	7
Wu et al., 2009 [26]	Han	AgP	Buccal mucosa	HB	31/35	48/60	72.9/50.0	0.04	17.89	7
Zhang et al., 2010 [39]	Han	CP	Buccal mucosa	HB	20~65	109/99	52.3/51.5	0.01	30.28	7
Qiu et al., 2013 [27]	Han	CP	Buccal mucosa	HB	46.4/37.2	60/81	65/51.9	0.91	22.01	7
Ma et al., 2013 [28]	Han	CP	Peripheral blood	HB	33~72	135/139	58.5/58.3	0.85	38.14	9
Ma et al., 2013 [28]	Hui	CP	Peripheral blood	HB	35~73	120/126	54.0/53.2	0.45	34.85	9

PvuII (rs2234693)										
Zhang et al., 2004 [19]	Han	AgP	Peripheral blood	PB	29.0 ± 6.3/29.1 ± 6.2	90/91	58.9/52.8	0.26	26.97	8
Zhang et al., 2004 [19]	Han	CP	Peripheral blood	PB	38.0 ± 10.4/29.1 ± 6.2	34/91	44.1/52.8	0.26	20.00	8
Wu et al., 2007 [23]	Han	CP	Buccal mucosa	HB	49/35	55/60	56.4/50.0	0.70	18.76	9
Wu et al., 2009 [26]	Han	AgP	Buccal mucosa	HB	31/35	48/60	72.9/50.0	0.70	17.89	8
Zhang et al., 2010 [39]	Han	CP	Buccal mucosa	HB	20~65	109/99	52.3/51.5	<0.01	30.28	7
Qiu et al., 2013 [27]	Han	CP	Buccal mucosa	HB	46.4/37.2	60/81	65/51.9	0.64	22.01	7
Ma et al., 2013 [28]	Han	CP	Peripheral blood	HB	33~72	135/139	58.5/58.3	0.52	38.14	9
Ma et al., 2013 [28]	Hui	CP	Peripheral blood	HB	35~73	120/126	54.0/53.2	0.23	34.85	9

CP: chronic periodontitis, AgP: aggressive periodontitis, HWE: Hardy-Weinberg equilibrium, PB: population-based, and HB: hospital-based.

^*∗*^Cases/controls.

^†^Assuming an odds ratio of 1.5 (small effect size) at *α* = 0.05 level of significance.

**Table 2:** Meta-analysis of the association between the ER-*α* gene XbaI polymorphism and periodontitis.

Genetic model and subgroup	Case/control	Heterogeneity		Test of association			*P* for Bon	*P* for FDR
		*P*	*I* ^2^ (%)	OR	95% CI	*P* for OR		
X versus x								
Overall	1514/1654	0.09	42.3	1.06	0.90, 1.24	0.480	0.480	0.48
CP	1238/1352	0.10	44.2	1.10	0.87, 1.40	0.417		
AgP	276/302	0.16	50.5	0.85	0.50, 1.45	0.552		
HWE (yes)	630/692	0.06	64.1	1.07	0.69, 1.66	0.779		
Han	1274/1402	0.09	43.6	1.10	0.93, 1.30	0.267		
Hui	240/252	NA	NA	0.84	0.56, 1.26	0.400		
Female	382/414	0.12	45.2	1.04	0.78, 1.40	0.773		
Male	316/388	0.62	0.0	0.64	0.45, 0.90	0.011		
XX versus xx								
Overall	485/449	0.41	3.6	1.67	1.13, 2.47	0.010	0.040	0.025
CP	413/384	0.23	26.6	1.71	1.12, 2.59	0.013		
AgP	72/65	0.86	0.0	1.44	0.48, 4.31	0.514		
HWE (yes)	196/224	0.19	69.0	0.79	0.36, 1.74	0.566		
Han	410/375	0.56	0.0	1.87	1.23, 2.84	0.003		
Hui	75/74	NA	NA	0.68	0.21, 2.26	0.533		
Female	117/77	0.21	32.5	3.08	1.34, 7.09	0.008		
Male	98/94	0.22	30.5	0.62	0.24, 1.60	0.326		
Xx versus xx								
Overall	661/777	0.01	60.9	0.72	0.51, 1.03	0.074	0.222	0.123
CP	532/632	0.01	64.0	0.75	0.50, 1.13	0.166		
AgP	129/145	0.06	72.5	0.63	0.24, 1.66	0.346		
HWE (yes)	304/330	0.10	57.1	1.17	0.70, 1.93	0.551		
Han	546/658	0.01	65.6	0.71	0.47, 1.07	0.099		
Hui	115/119	NA	NA	0.83	0.49, 1.39	0.479		
Female	159/199	0.45	0.0	0.46	0.30, 0.71	0.001		
Male	152/161	0.92	0.0	0.51	0.32, 0.80	0.003		
XX + Xx versus xx								
Overall	757/827	0.05	47.9	0.88	0.72, 1.07	0.202	0.404	0.253
CP	619/676	0.07	48.0	0.91	0.66, 1.25	0.561		
AgP	138/151	0.08	68.5	0.68	0.28, 1.64	0.392		
HWE (yes)	315/346	0.06	64.4	1.14	0.67, 1.95	0.638		
Han	637/701	0.03	54.1	0.87	0.62, 1.21	0.409		
Hui	120/126	NA	NA	0.81	0.49, 1.34	0.416		
Female	191/207	0.44	0.0	0.62	0.41, 0.95	0.027		
Male	158/194	0.88	0.0	0.52	0.33, 0.80	0.003		
XX versus Xx + xx								
Overall	757/827	0.17	30.9	2.07	1.43, 3.00	0.001	0.005	0.005
CP	619/676	0.08	47.1	2.13	1.44, 3.17	<0.001		
AgP	138/151	0.85	0.0	1.67	0.58, 4.82	0.342		
HWE (yes)	315/346	0.32	13.0	0.76	0.35, 1.66	0.489		
Han	637/701	0.31	14.9	2.33	1.57, 3.47	<0.001		
Hui	120/126	NA	NA	0.74	0.23, 2.40	0.614		
Female	191/207	0.17	38.1	4.79	2.16, 10.61	<0.001		
Male	158/194	0.22	30.8	0.83	0.33, 2.10	0.694		

CP: chronic periodontitis, AgP: aggressive periodontitis, HWE: Hardy-Weinberg equilibrium, NA: not available, Bon: Bonferroni test, and FDR: false discovery rate.

**Table 3:** Meta-analysis of association between ER-*α* gene PvuII polymorphism and periodontitis.

Genetic model and subgroup	Case/control	Heterogeneity		Test of association			*P* for Bon	*P* for FDR
		*P*	*I* ^2^ (%)	OR	95% CI	*P* for OR		
P versus p								
Overall	1302/1494	0.66	0.0	1.06	0.91, 1.23	0.452	0.904	0.565
CP	1026/1192	0.43	0.0	1.05	0.89, 1.25	0.549		
AgP	276/302	0.77	0.0	1.08	0.78, 1.50	0.632		
HWE (yes)	1084/1296	0.55	0.0	1.07	0.91, 1.26	0.428		
Han	1062/1242	0.59	0.0	1.04	0.88, 1.23	0.681		
Hui	240/252	NA	NA	1.17	0.82, 1.67	0.374		
PP versus pp								
Overall	334/431	0.96	0.0	1.01	0.75, 1.37	0.927	0.927	0.927
CP	268/348	0.88	0.0	0.98	0.70, 1.39	0.926		
AgP	66/83	0.78	0.0	1.13	0.59, 2.18	0.707		
HWE (yes)	257/361	0.92	0.0	1.01	0.72, 1.43	0.937		
Han	286/361	0.97	0.0	0.95	0.68, 1.33	0.782		
Hui	48/70	NA	NA	1.37	0.65, 2.86	0.405		
Pp versus pp								
Overall	523/587	0.01	61.1	1.29	0.86, 1.93	0.220	1.000	0.555
CP	415/471	<0.01	72.1	1.24	0.73, 2.13	0.430		
AgP	108/116	0.94	0.00	1.41	0.82, 2.43	0.219		
HWE (yes)	444/515	0.01	65.1	1.33	0.84, 2.12	0.225		
Han	428/492	0.02	60.3	1.18	0.76, 1.83	0.454		
Hui	95/95	NA	NA	2.18	1.17, 4.06	0.014		
PP + Pp versus pp								
Overall	651/747	0.05	50.4	1.23	0.88, 1.71	0.222	0.888	0.555
CP	513/596	0.02	64.4	1.20	0.78, 1.86	0.414		
AgP	138/151	0.94	0.0	1.31	0.79, 2.19	0.296		
HWE (yes)	542/648	0.04	55.6	1.27	0.86, 1.86	0.226		
Han	531/621	0.07	49.0	1.15	0.80, 1.63	0.453		
Hui	120/126	NA	NA	1.89	1.05, 3.41	0.035		
PP versus Pp + pp								
Overall	651/747	0.89	0.0	0.88	0.68, 1.15	0.358	1.000	0.565
CP	513/596	0.73	0.0	0.87	0.65, 1.18	0.385		
AgP	138/151	0.71	0.0	0.91	0.52, 1.59	0.744		
HWE (yes)	542/648	0.84	0.0	0.85	0.64, 1.15	0.299		
Han	531/621	0.82	0.0	0.90	0.67, 1.21	0.499		
Hui	120/126	NA	NA	0.81	0.44, 1.47	0.481		

CP: chronic periodontitis, AgP: aggressive periodontitis, HWE: Hardy-Weinberg equilibrium, NA: not available, Bon: Bonferroni test, and FDR: false discovery rate.
