# Supplementary material for: Association between Estrogen Receptor-α Gene XbaI and PvuII Polymorphisms and Periodontitis Susceptibility: A Meta-Analysis
Source: Dis Markers. 2015 Nov 24;2015:741972. doi: 10.1155/2015/741972 (PMC4672125; doi:10.1155/2015/741972)
Supplement: Supplementary file 1 — The scale for quality assessment is in supplementary Table 1. [file 741972.f1.pdf]

Supplementary table 1.

Scale for quality assessment

Criteria	Score
**Representativeness of cases**	
Consecutive/randomly selected from case population with clearly defined sampling frame	2
Consecutive/randomly selected from case population without clearly defined sampling frame or with extensive	1
Not described	0
**Source of controls**	
Healthy- or population-based	2
Hospital-bases	1
Not described	0
**Hardy-Weinberg equilibrium in controls**	
Hardy-Weinberg equilibrium	2
Hardy-Weinberg disequilibrium	1
**Genotyping examination**	
Genotyping done under "blinded" condition	1
Unblinded done or not mentioned	0
**Association assessment**	
Assess association between genotypes and periodontitis with appropriate statistics and adjustment for confounders	2
Assess association between genotypes and periodontitis with appropriate statistics and without adjustment for confounders	1
Inappropriate statistics used	0
**Total sample size**	
≥200	2
>100 but <200	1
≤100	0
